# Volunteers’ Support of Carers of Rural People Living with Dementia to Use a Custom-Built Application

**DOI:** 10.3390/ijerph18189909

**Published:** 2021-09-20

**Authors:** Clare Wilding, Hilary Davis, Tshepo Rasekaba, Mohammad Hamiduzzaman, Kayla Royals, Jennene Greenhill, Megan E. O’Connell, David Perkins, Michael Bauer, Debra Morgan, Irene Blackberry

**Affiliations:** 1 John Richards Centre for Rural Ageing Research, La Trobe University, Wodonga 3689, Australia; c.wilding@latrobe.edu.au (C.W.); t.rasekaba@latrobe.edu.au (T.R.); k.royals@latrobe.edu.au (K.R.); 2 Centre for Social Impact, Swinburne University, Melbourne 3122, Australia; hdavis@swin.edu.au; 3 Department of Rural Health, The University of Newcastle, Callaghan 2430, Australia; khoka.hamiduzzaman@newcastle.edu.au; 4 College of Medicine and Public Health, Flinders University, Renmark 5341, Australia; jennenegreenhill@gmail.com; 5 Department of Psychology, University of Saskatchewan, Saskatoon, SK S7N 5E4, Canada; megan.oconnell@usask.ca; 6 Centre for Rural and Remote Mental Health, The University of Newcastle, Orange 2800, Australia; david.perkins@newcastle.edu.au; 7 Australian Centre for Evidence Based Aged Care, La Trobe University, Melbourne 3083, Australia; m.bauer@latrobe.edu.au; 8 Department of Medicine, Canadian Centre for Health & Safety in Agriculture, University of Saskatchewan, Saskatoon, SK S7N 2Z4, Canada; debra.morgan@usask.ca

**Keywords:** rural, dementia, older adults, carers, online technology, volunteer, qualitative research, digital literacy

## Abstract

There is great potential for human-centred technologies to enhance wellbeing for people living with dementia and their carers. The Virtual Dementia Friendly Rural Communities (Verily Connect) project aimed to increase access to information, support, and connection for carers of rural people living with dementia, via a co-designed, integrated website/mobile application (app) and Zoom videoconferencing. Volunteers were recruited and trained to assist the carers to use the Verily Connect app and videoconferencing. The overall research design was a stepped wedge open cohort randomized cluster trial involving 12 rural communities, spanning three states of Australia, with three types of participants: carers of people living with dementia, volunteers, and health/aged services staff. Data collected from volunteers (*n* = 39) included eight interviews and five focus groups with volunteers, and 75 process memos written by research team members. The data were analyzed using a descriptive evaluation framework and building themes through open coding, inductive reasoning, and code categorization. The volunteers reported that the Verily Connect app was easy to use and they felt they derived benefit from volunteering. The volunteers had less volunteering work than they desired due to low numbers of carer participants; they reported that older rural carers were partly reluctant to join the trial because they eschewed using online technologies, which was the reason for involving volunteers from each local community.

## 1. Introduction

Family carers provide vital personal, socio-economic, and functional support to people living with dementia, but carers themselves need support to provide this care [1,2]. Due to the way dementia affects a person’s cognitive capacity and daily living activities, carers may have to provide physical, psychological and emotional care [3,4]. Such extensive involvement of care provision places carers at risk of social isolation and diminished social networks [5], for example giving up holidays and hobbies and spending less time with family and friends [6]. In addition to loneliness and isolation, Australian rural carers face other challenges in obtaining the support they need. Rural areas frequently lack health and social services to help them manage dementia, absence of public transport makes accessing services challenging, and social stigma also restricts carers from accessing support services for themselves [6,7]. Increased engagement with services and better connection to support networks for carers may improve their health outcomes. 

In Australia, the support and services available for carers of a person living with dementia generally include nursing care, personal counselling, domestic help, allied health support and respite care programs [7,8]. These programs have demonstrated success in reducing carers’ levels of depression and distress and improving wellbeing. For example, randomized-control studies, such as Tremont et al.’s (2015) therapeutic counselling (that offers dementia education, emotional support and teaching coping strategies) in the USA and Chen et al.’s (2015) training program (providing problem solving skills, knowledge of dementia, social resources and emotional support) were found effective in improving carers’ mental health and quality of life [9,10]. Similar services are often limited in rural areas of Australia and engaging rural carers can be challenging [6]. 

Using online technologies to provide services to rural carers may help overcome lack of services and improve access to services in a large geographical area. A systematic review and meta-analysis of remotely delivered education for carers of people living with dementia demonstrated positive impacts for carer wellbeing [11]. O’Connell et al. (2021) described how telephone-based training could help older adults and carers use internet-based methods for videoconferencing to maintain social and community connections during the global pandemic [12]. 

Unfortunately, rural carers are restricted in their ability to use information and communication technologies (ICT) due to lack of rural technology infrastructure [13]. The rural paradox, as described by Salemink and colleagues (2017) [13], refers to the lack of access to ICT infrastructure for those who would benefit from it most, that is, rural residents. Lack of exposure to technology due to ICT barriers creates an additional psychological barrier to technology adoption, which is referred to as the double-digital divide [14]. O’Connell et al. (2018) argued that physical and psychological factors in the rural context can create barriers to technology adoption and sustained technology use but that training and support appears to mitigate some of the psychological barriers to technology adoption [15].

The Verily Connect app was co-designed with carers and key stakeholders (rural health service providers, technology specialists, representatives from Carers Australia and Dementia Australia) to provide additional support for carers of people living with dementia. Volunteers who were trained to help the carers access the online support were an integral part of the Verily Connect project. As part of a larger randomized stepped wedge cluster trial, qualitative data about the experience of volunteering and feedback from volunteers were evaluated and reported here. 

## 2. Materials and Methods

The design of the Verily Connect project was an open cohort stepped wedge cluster randomized controlled trial [16]. A cluster randomized trial design was selected because the study involved multiple participant groups (carers, service providers and volunteers) in a community setting, and each participant group had a different role. Twelve rural communities (the clusters) were recruited through the research team’s existing networks. There were 8 participating communities in Victoria, and 2 each in New South Wales and South Australia. Three clusters commenced the Verily Connect intervention every 8 weeks (commencing August 2018 and concluding March 2019), while the remaining communities continued in the waiting control phase, until all 12 were engaged in the intervention. (More details about the methods can be found in the Australian New Zealand Clinical Trials Registry, ACTRN12618001213235.) 

Volunteers were recruited at meetings held in each community to introduce and describe the Verily Connect Project. Some participants were existing volunteers with a community organization such as a library or volunteer organization and they agreed to take on an additional volunteering role for the Verily Connect Project. Volunteers were also recruited via advertising in local community newsletters or the local volunteer resource service. 

The role of the volunteers was to undertake training and support carers in their local community to use the Verily Connect app and Zoom videoconferencing technology. Research staff matched volunteers with carers. Volunteers supported carers to log in and navigate the Verily app. They assisted carers to join videoconferenced carer peer support meetings. For example, volunteers conducted practice videoconference meetings to build carers’ confidence in using Zoom and to solve problems if required. In some communities, the volunteers took on an additional role of promoting Verily Connect, for example by distributing project flyers and speaking to community groups. 

The volunteer training program was adapted from training designed for The Rural Dementia Volunteers Program [17]. The Verily Connect training programme had a technology focus and was developed and delivered by the second author, with support from the larger research team. The training program was one day in length, with an optional drop-in session the following day. Typically, the drop-in session was used to address technological-focused issues, such as connectivity concerns and to give volunteers additional practice using the Verily Connect app and videoconferencing technology. 

The training was delivered once in each of the 12 participating communities, within the first 1–3 weeks of the intervention phase for the community. Local community facilities provided venues for the training, for example at a health service, neighbourhood house or library. During their training, the volunteers were provided with online resources and practical handouts including a copy of the Volunteer Training PowerPoint slides, a Volunteer Handbook (including a role description and code of conduct), and Dementia Australia resources such as The Dementia Guide [18]. The Dementia Guide is a free resource for people diagnosed with or impacted by dementia. The resource outlines the emotional impact of dementia, available medical treatment, and support and services available. It also includes information about living well with dementia and making plans for the future. 

### Training Program Content

After introductions, the benefits of volunteering for volunteers were discussed; these benefits included increased knowledge of memory loss and support services, increased sense of connectedness and social networks, and learning about online technologies. Possible benefits for carers (such as increased capacity to cope with caring and increased social support) and communities (such as increased community capacity to support people with memory loss, better community resilience and increased capacity to utilise online technologies) were also discussed. 

The training program included information about the signs of dementia, based on Dementia Australia resources [19]. In each community, relevant local experts attended the training sessions, including representatives from Dementia Australia and participating health services. These people helped to clarify memory loss and behaviours associated with dementia. 

The importance of listening and using empathy with carers was highlighted, for example through discussing that depression, loneliness and social isolation can occur when caring for a person living with dementia [20] (pg. 2614). International speaker, Heather Plett’s blog on “Holding space” [21] was used to illustrate that the volunteer’s role in the Verily Connect project was to be supportive without trying to take charge or ‘fix’ problems. A self-assessment listening exercise designed by Garber [22] (p. 37) was used as a tool for reflection on individual listening styles. 

In a later part of the training, the volunteers explored how to use the Verily Connect website and app using a Bring Your Own Device (BYOD) session. During this part of the training, volunteers downloaded the Verily Connect app, practiced logging in and out, and explored the different functions of the app using their laptop, tablet, or mobile phone. The volunteers particularly enjoyed exploring service availability in their community, located in a Google map-based feature in the Verily Connect app. Volunteers provided local knowledge of additional services such as transport options and community activities, which was instrumental for updating local service provision options. The volunteers also learned how to use the in-app chat and how to operate the videoconferencing software, Zoom. The training also addressed online communication including online etiquette and safety, such as not disclosing personal or sensitive information.

Data collected from volunteers was used for obtaining feedback about being a volunteer in the Verily Connect project and about the Verily Connect app. During the implementation phase, the research team members provided short written memos of their reflections on volunteer support videoconference meetings (20 memos), and personal communications (mostly email and telephone) (55 memos); a total of 75 memos were collected. At the end of the trial period, 5 focus groups and 8 individual interviews with volunteers were completed, audio-recorded, and transcribed verbatim. 

Qualitative data (memos, transcripts of focus groups and interviews) were analyzed inductively using methods described by Miles and Huberman (1994) [23], Stanley (2015) [24], and Streubert and Carpenter (1995) [25]. The collected data were imported into NVivo and coded line by line, using a descriptive framework: what was valuable and what was challenging about being a volunteer and what aspects of the Verily Connect project worked well and what needed improving. Additional categories arose inductively, such as reflections about challenges for carers that limited their recruitment to the study and technology challenges experienced by volunteers and carers. Once coded and categorized, further iterative inductive analysis was used to collapse categories and build themes. 

## 3. Results

A total of 39 volunteers were enrolled across 12 communities. Their ages ranged from 22 to 78 and the median age was 66 years. Most volunteers were female (82%). There were four main themes regarding the volunteers’ perspectives of participation in Verily Connect: (1) ease of use; (2) benefits of volunteering for Verily Connect; (3) the biggest challenges for volunteers; and (4) technological challenges. 

### 3.1. Verily Connect App Was Easy to Use

The volunteers found the Verily Connect app to be easy to use: “I quite like the app… It can be used on a smart phone which is very helpful because … a lot of people don’t have computers but they’re quite happy to work on their phone. …It’s very easy to use, which I can’t say for a lot of apps [laughs], but the Verily app is actually very easy to use” (Interview 4). Another volunteer noted that the technology was suitable even for people with a beginner level of understanding: “It was aimed at people who probably weren’t completely, well let’s face it, for that sort of technology it’s a relatively new thing, and it was aimed at people who wouldn’t have grown up with it. I thought it worked really well. It works, it was self-explanatory, quite clear. Yeah, it was good” (Interview 6). The volunteers reported that carers were able to quickly learn how to use the app: “The lady we showed cottoned onto it fairly quickly - didn’t have any trouble - and she was using it on her phone” (Interview 7). 

### 3.2. Benefits of Volunteering

The Verily Connect volunteers valued the opportunity to work with and help carers. They reported that they received benefit from their volunteering and had the opportunity to learn about dementia and about different types of technology. They valued the training and they derived support from the research team and each other as they participated in videoconference support meetings. They appreciated the opportunity to help improve the app by providing feedback based on their local knowledge. Sub-themes about benefits of being a volunteer are illustrated in Table 1.

### 3.3. Lack of Work for Volunteers Was a Significant Challenge

The main challenge for volunteers was the low participation rates of carers, which meant that volunteers were unable to completely fulfil their volunteering role because there were not enough carers who wanted their assistance. There were more volunteers (*n* = 39) than carers (*n* = 37) and not all carers needed or wanted help with technology. Therefore, some volunteers were not matched with carers. (The carers’ reluctance to use technology is addressed in the next section.) Some volunteers tried to increase recruitment of carer participants by distributing flyers and telling others in their community about the project. The volunteers expressed regret and disappointment about the low numbers of carer participants and the resulting lack of need for volunteer services (Table 2).

### 3.4. Challenges Relating to Technology

Volunteers noted that many older carers who they invited to participate in Verily Connect appeared to have been discouraged from participating due to not feeling comfortable or confident with the thought of using online technology. This unease with technology in general stopped many carer participants from even trying the Verily Connect intervention (even though volunteers were available to provide help in using the app and with videoconferencing). “I think that phobia of the computers and stuff is a big thing with the older population” (Focus Group 2). “For a lot of elderly people … it’s just convincing them that they’re not going to crash the computer … it’s getting confidence in using IT [information technology]” (Focus Group 3). 

Another challenge was lack of or poor Internet connectivity in rural areas. This was another issue that resulted in carers deciding that Verily Connect was not for them, even without attempting to get involved. “In the country your connectivity is not always brilliant” (Focus Group 2). “The internet’s sort of not all that good at times … it’s only a very small town where she [a carer] is” (Focus Group 1). 

Some of the volunteers were themselves older people; they considered that sometimes older people struggle with learning. They felt they were novices at using technology:

“We’re a small town and … these people are in their eighties that I’ve been in contact with. The internet’s sort of not all that good at times. It’s also the use of a computer, or a phone or an iPad. They just feel it’s beyond their capabilities at their age. I don’t know, maybe someone younger may definitely come on board … Well, I’ve still got my “L plates” on when it comes to technology anyway, and a lot of older people have around here” (Focus Group 1).

“I’ve got kids in their 30s now and they have no problems whatsoever with technology. None whatsoever. But … that’s a big difference between them and, say, my generation or a bit older. I was never involved in a job that really needed too many computer skills… It’s a valuable thing, obviously you folk can see it and everybody … involved in it would see the value of it. But unfortunately, I think you’re a little ahead of my generation…. It just feels as though it’s a little bit ahead of its time” (Interview 6).

## 4. Discussion

Given the high numbers of people living with dementia and the lack of services and access to services in rural Australia, it is necessary to find innovative ways to address gaps in dementia service provision for people living with dementia and their carers. Carers are conscious of their need for support but they have few options [1,2]. Improving access to information, dementia training and carer support have been identified as key priorities in rural areas [6]. Therefore, the finding that volunteers experienced the Verily Connect app was easy to use is heartening. If volunteers, who are lay members of society, find the app informative and easy to use, it is anticipated that other app users (including carers and service providers) may similarly find the app to be user-friendly. Services to support carers that use information and communication technologies, such as Verily Connect, may assist in overcoming some of the longstanding service shortages and access difficulties that have disadvantaged rural carers.

Verily Connect volunteers valued the training and support provided. Rural health services have limited capacity to design and deliver training opportunities and can experience many obstacles to accessing training, including the time training takes and distances to travel for training. Verily Connect training was provided onsite in the local community and ongoing support for volunteers was provided online. This type of hybrid training and support may assist organizations that struggle to release staff and find a replacement and cannot afford the cost of the training and the payment of the staff for their time. This research offers a solution for overcoming the tyranny of distance by creating a local pool of trained volunteers, who receive additional remote mentoring. Such a model blends the advantages of face-to-face support for carers, with lower cost and increased access to support for volunteers.

Participation in the Verily Connect project increased workforce capacity and support in rural communities by providing volunteers embedded in the local community who had improved knowledge of dementia and available local support services, and increased skill in using technology. Because of their involvement in Verily Connect, volunteers developed their own competence and confidence in using technology and they were then available to support carers. Other studies [13] have similarly identified the value of learning about dementia using technology to improve ability to access information and develop confidence and ability to use communication technology. 

This research enabled volunteers to be actively involved in improving the program by providing feedback. These findings build upon the positive impacts of other effective training programs that provide problem solving skills, social resources, and emotional support, and improve knowledge of dementia that can result in improving carers’ mental health and quality of life [9,10]. Thus, this approach to co-designing peer support and information packages is highly recommended and it has been very effective in other research [26].

The apparent reticence of older carers to trying web-based technology is worthy of further investigation. It is unknown if even fewer carer participants would have enrolled without the availability of volunteer help. Thus, research that compares implementation using volunteers with implementation without volunteers, particularly in rural settings, could be valuable. As this study was completed prior to the COVID-19 global pandemic, it is also unknown if uptake of the Verily Connect technology may be more attractive to carer participants in a post-COVID world, which has necessitated the use of technologies to enable access to many services and even social contact. 

### Study Strengths and Limitations

There was significant data triangulation in this study, as data were collected from volunteers and researchers across multiple time points and via multiple modes (including face-to-face, videoconference, and telephone interviews and focus groups). The data were analyzed and reviewed by a group of very experienced researchers. 

As noted by the volunteers, the main limitation of the study was the low numbers of carer participants. Although a wide variety of recruitment strategies was tried, higher numbers of carer participants could not be recruited. Such a challenge has been encountered in similar research in rural communities [26]. 

## 5. Conclusions

It is challenging for rural Australian carers of people living with dementia to receive the support they need. The Verily Connect project trialed the use of human-centered technologies as a means of increasing support, and volunteers were trained to help carers with using the technologies. There were benefits to volunteers in being involved in this project, even though many of the volunteers were underutilized due to low enrollments of carer participants. Although there is great promise for using human-centered technologies to increase support for carers, there were significant challenges in convincing carers to even try using the technology, even with the ready support of locally available volunteers. It may be that in future volunteers are not needed to assist people to learn how to use technology such as the Verily Connect app and videoconferencing, but rather volunteers may be needed as champions to inspire and motivate carers to give human-centered technologies a try. 

## Figures and Tables

**Table 1:** Benefits of being a Verily Connect volunteer.

Sub-themes	Supporting Data
Working with and helping carers	“It was really good to be able to, the lady that I helped … just being able to help her. I think for her it was that little leg up to get some more support.” (Focus Group 3)
Learning about dementia and technology	“Learning something new—Zoom. I’d never used that before. So that was good. That was interesting.” (Focus Group 2) “We used it for learning, and we did actually pick up a lot of information on dementia.” (Interview 7)
Training and support provided by Verily Connect	“I’d agree that the training worked very well. That I felt at the end of it that I was quite confident and comfortable about what the role was and what the resources available were that we were to show people. I thought it was very well done. It was a good balance between just dementia and general information as well as the technology side and the Verily [Connect] side.” (Focus Group 4)
	“The Zoom conference, that was really interesting because we also had people from Victoria as well as Victor Harbor. It was really interesting to hear their comments and how they’d managed to interact with carers of people with dementia and the help they’ve been able to give them.” (Focus Group 4)
Providing feedback to improve the app	“I don’t know that there’s much to improve on the app, apart from getting locations of medical centers and whatever a bit more accurate.” (Interview 2)

**Table 2:** Challenge caused by low numbers of carer participants.

Volunteers’ Comments about Low Carer Participation Rates
“The volunteer is disappointed she hasn’t been able to do much—she has interacted with two people in their 80s who she feels would be ideal for the program, however one is not comfortable with technology and the other is too busy to participate.” (Memo 64)
“The difficulty about being a volunteer in this project is we’ve actually not had to do anything as yet, which is a bit disappointing. But I guess that comes about because being a small community, people have people that help them. A lot of people … actually fall back on their friends and family because it’s such a tight knit area, that they don’t really look for outside help.” (Interview 4)
“The challenge is that … we couldn’t get people to take it up, I guess. We put out leaflets, we advertised. We had things up at the doctors… It just didn’t take off. I think that was the biggest challenge for me. (Focus Group 1)

## Data Availability

The data presented in this study are available on request from the corresponding author. The data are not publicly available due to privacy of participants.
